# Extraction, characterization and anti-oxidant activity of polysaccharide from red *Panax ginseng* and *Ophiopogon japonicus* waste

**DOI:** 10.3389/fnut.2023.1183096

**Published:** 2023-05-24

**Authors:** Jia Kang, Jue Zhao, Lan-Fang He, Li-Xia Li, Zhong-Kai Zhu, Meng-Liang Tian

**Affiliations:** ^1^Hospital of Chengdu University of Traditional Chinese Medicine, Chengdu, China; ^2^Natural Medicine Research Center, College of Veterinary Medicine, Sichuan Agricultural University, Chengdu, China; ^3^College of Agronomy, Sichuan Agricultural University, Chengdu, China

**Keywords:** red *Panax ginseng*, *Ophiopogon japonicus*, waste, polysaccharide, prebiotics, antioxidants

## Abstract

Red ginseng and *Ophiopogon japonicus* are both traditional Chinese medicines. They have also been used as food in China for thousands of years. These two herbs were frequently used in many traditional Chinese patent medicines. However, the carbohydrate compositions of these two herbs were not normally used during the production of said medicine, such as Shenmai injection, resulting in a large amount of waste composed of carbohydrates. In this study, the extraction conditions were optimized by response surface methodology. The Shenmai injection waste polysaccharide was extracted by using distilled water that was boiled under the optimized conditions. The Shenmai injection waste polysaccharide (SMP) was thereby obtained. SMP was further purified by anion exchange chromatography and gel filtration. With this method, a neutral polysaccharide fraction (SMP-NP) and an acidic polysaccharide fraction (SMP-AP) were obtained. The results of structure elucidation indicated that SMP-NP was a type of levan, and SMP-AP was a typical acidic polysaccharide. SMP-NP exhibited potential stimulation activity on the proliferation of five different *Lactobacilli* strains. Therefore, SMP-AP could promote the antioxidant defense of IPEC-J2 cells. These findings suggest that Shenmai injection waste could be used as a resource for prebiotics and antioxidants.

## 1. Introduction

Shenmai San, which consists of *Panax ginseng* and *Ophiopogon japonicus*, is an ancient Chinese patent medicine that first appeared in “Medical Origins.” It is used to treat diseases like heart attacks, congestive heart failure, and severe bronchitis induced by *Qi* and *Yin* deficiency (1). In modern China, Shenmai San has been developed into an injection preparation (Shenmai injection) that is a sterile aqueous solution prepared by the combination of Red ginseng ethanol extract and *O. japonicus* ethanol extract. Clinical pharmacy studies reveal that the Shenmai injection is more effective when used to treat cardiovascular diseases, such as coronary heart disease, viral myocarditis, and chronic pulmonary heart disease (1, 2). It is often combined with chemotherapeutic drugs to increase their curative effects and improve the immune function of cancer patients (3). Saponin ingredients from *P. ginseng* and *O. japonicus* are the major components of the Shenmai injection (4). And yet, the carbohydrate composition of these two herbs is not used during the production of the Shenmai injection, resulting in a large amount of waste composed of carbohydrates. Due to the significant increase in the production of Shenmai injection in China, the development and utilization of waste based on its active carbohydrate ingredients would have economic and environmental benefits.

*Panax ginseng* is a traditional Chinese medicinal herb. For thousands of years, it has been used to increase vitality and boost the immune system. Multiple bioactive ingredients like ginsenosides, phytosterols, and polyacetylenes are isolated from *P. ginseng*, but polysaccharides are thought to be one of the most important ingredients due to their effective bioactivities (5, 6). Similar to *P. ginseng*, *O. japonicas* is important in traditional Chinese medicine. It has been used to nourish the *Yin*, promote body fluid production, and treat lung diseases. 71% of *O. japonicus* is made up of carbohydrates (7). So, it is not a surprise that the polysaccharides from it can be used for several bioactivities, such as anti-diabetes, antioxidants, and improved immunity (8, 9). While lots of studies reported the isolation and characterization of polysaccharides from *P. ginseng* and *O. japonicus* respectively, the polysaccharides from the combined extraction of these two medicinal herbs have not been well studied.

In this study, we aimed to perform single-factor extraction experiments and optimize them to isolate polysaccharides from Shenmai injection waste. We followed this with anion exchange chromatography and gel filtration to obtain the neutral and acidic polysaccharide fractions. Size exclusion chromatography, methanolysis, and NMR were performed to determine the molecular weight, monosaccharide composition, and glycosidic linkage of these polysaccharides. Finally, the prebiotic and antioxidant activities were analyzed by *in vitro* experiments.

## 2. Materials and methods

### 2.1. Materials and reagents

The dried red *Panax ginseng* and *Ophiopogon japonicus* residues from Shenmai injection production were obtained from Ya’an Sanjiu Pharmaceutical Co., Ltd. (Ya’an City, Sichuan Province, China). Both herbs were identified by Li-Xia Li of Sichuan Agriculture University. Specimens were deposited in the College of Veterinary Medicine, Sichuan Agricultural University.

DEAE-cellouse and Agarose gel 6FF were obtained from Beijing Ruidahenghui Science & Technology Development Co., Ltd. The MRS medium (HB0384-1), peptone (HB8276), and tryptone (HB8270) were purchased from Hopebio Biotechnology Co., Ltd. (Qingdao, China). The yeast extract powder (JM-500) was purchased from Biotopped Science and Technology Co., Ltd. (Beijing, China). The McIntosh Turbidimetric tube (G60346) was obtained from Wenzhou Kangtai Biotechnology Co., Ltd. (Zhejiang, China). The standard fructooligosaccharide (QHT-FOS-P95S) and inulin (Orafti^®^HP) were purchased from Quantum Hi-Tech Biological Co., Ltd. (Jiangmen, China) and Beneo-Orafti (Belgium), respectively.

The standard of fructose (Fru) and glucose (Glc) were purchased from Solarbio (Beijing, China). All other chemicals, such as phenol, sulfuric acid, acetone, boric acid, glycerin, etc., were of analytical grade and obtained from the Chengdu Kelong chemical factory (Chengdu, China).

### 2.2. Extraction and determination of polysaccharide from Shenmai injection waste

The powdered red *P. ginseng* and *O. japonicus* were mixed with a ratio of 1:1(w/w) and isolated using distilled water and different extraction conditions. The aqueous extracts were collected and concentrated by Rotary Evaporator (Shanghai Yarong Biochemical Instrument Factory Co., Ltd). Four times the volume of ethanol was poured into the water extracts and placed at 4°C for 24 h. The mixture was centrifuged (3,500 rpm, 10 min), and the insoluble residue was separated. The polysaccharide was obtained after lyophilization. The content of the carbohydrate was determined by the phenol-sulfuric acid method (10). The extraction yield of the polysaccharide was calculated according to the content of the carbohydrate.

### 2.3. Design of extraction conditions

#### 2.3.1. Single-factor experiment

The optimum extraction conditions of polysaccharides from Shenmai injection waste were measured by single-factor experiments and the response surface method (RSM). The single-factor experiment was executed in a designed extraction time (ranging from 0.5–3 h), a designed extraction temperature (ranging from 50°C–100°C), and with an extraction ratio of solvent to material (ranging from 10:1–50:1) with 1.0 g of red ginseng and *O. japonicus* powder mixture (after ethanol extraction) (11, 12). One factor was kept invariable for each study and each group in triplicate. After extraction, the aqueous extracts were centrifuged and freeze-dried. Then the powder was dissolved into 1 mg/mL, and the carbohydrate component was determined by the method described above.

#### 2.3.2. Optimization of extraction conditions by BBD

Box–Behnken design (BBD) is a type of response surface design. It is an independent quadratic design in that it does not contain an embedded factorial or fractional factorial design. In this design the treatment combinations are at the midpoints of edges of the process space and at the center. These designs are rotatable (or near rotatable) and require 3 levels of each factor. It is more efficient and easier to arrange and interpret experiments in comparison with others (13). Based on the single-factor experiment described above, BBD experiment was adopted and revealed in Table 1 with three-level-three-factors. Those factors were mentioned above. The extraction temperature (*X*_1_), the ratio of solvent to material (*X*_2_), and the extraction time (*X*_3_) were designed using SAS. JMP. 13.0 software (Statistical analysis system, United States).

**Table 1:** Central composite design and the extraction yield of polysaccharide.

Runs	Independent variables			Uncoded level			Extraction yield/%
	Coded level						
	*X*_1_	*X*_2_	*X*_3_	*X*_1_	*X*_2_	*X*_3_	
1	0	1	−1	90	1:40	20	51.3 ± 0.396
2	0	−1	−1	90	1:20	20	52.14 ± 3.43
3	−1	0	1	80	1:30	40	57.75 ± 0.1904
4	0	0	0	90	1:30	30	60.37 ± 3.274
5	−1	−1	0	80	1:20	30	51.93 ± 2.593
6	−1	0	−1	80	1:30	20	60.6 ± 1.5132
7	0	1	1	90	1:40	40	60.12 ± 1.35
8	0	0	0	90	1:30	30	62.67 ± 1.355
9	1	0	1	100	1:30	40	60.6 ± 1.355
10	0	−1	1	90	1:20	40	60.78 ± 1.047
11	1	−1	0	100	1:20	30	61.36 ± 2.079
12	1	0	−1	100	1:30	20	60.45 ± 0.997
13	1	1	0	100	1:40	30	60.75 ± 0.467
14	0	0	0	90	1:30	30	60.56 ± 1.817
15	−1	1	0	80	1:40	30	61.69 ± 0.9405

The variables were coded according to the following formula:


$$X_{i}=\frac{X_{i}-X_{0}}{\Delta x}$$

where *X_i_* is the coded value of the variable *X_i_*, *X*_0_ is the value of *X_i_* at the central point, and Δ*x* is the amplitude of variation. The results were analyzed and fitted to a second-order polynomial model.

In the formula, *Y* is the response variable (the extraction yield of polysaccharide). *A*_0_, *A_i_*, *A_ii_*, and *A_ij_* are the intercept linear, quadratic, and interaction coefficients of *X*_1_, *X*_2_, and *X*_3_, respectively. *X_i_* and *X_j_* are the coded independent variables, and the terms of *X_i_*^2^ represent the quadratic terms. Analyses of the variance were evaluated *via* the ANOVA procedure. The fitness of this predictive model was performed by the coefficient of determination *R*^2^ and the adjusted-*R*^2^. Then the statistical significance and regression coefficients were checked using the *F*-test at a probability (*p*) of 0.01 or 0.05.

### 2.4. DEAE-cellouse ion exchange chromatography

300 mg of crude polysaccharide was dissolved with 20 mL of distilled water and filtered with a 0.45 μm filter. Then the polysaccharide solution was injected into the DEAE-cellouse ion column (50 mm × 40 cm, Beijing RuiDaHengHui Science & Technology Development Co., Ltd.), and distilled water was used as an elution buffer. The neutral fraction was eluted with a three-fold column volume of distilled water at the speed of 2 mL/min, combining the phenol-sulfuric acid method (14). We collected all the elution, concentrated it, and then preserved the solution *via* lyophilization, named SMP-NP. The column was further eluted using a gradient elution of NaCl (0–1.5 M), and the acidic fraction SMP-AP was obtained.

### 2.5. Molecular weight determination

Molecular weight was measured by size exclusion chromatography. Five types of dextrans were used as standard (10, 70, 200, 800, and 1,000 kDa). 2 mg of each of the standard dextrans were weighted, respectively. Then all standard dextrans were mixed and dissolved in a 10 mmol/L NaCl solution and filtrated *via* a 0.22 μm filter. The mixture solution was then injected into the column and eluted with a 10 mmol/L NaCl solution at a speed of 0.2 mL/min. We kept 1 mL in volume per tube. After that, we collected all elution. The carbohydrate fraction was determined using the phenol-sulfuric acid method. We calculated the linear relationship between the molecular weight logarithm of the glucan and the elution volume and obtained the molecular weight of the carbohydrate fraction.

### 2.6. Chemical compositions and linkage determination

The SMP-AP was subjected to methanolysis with 3 M of hydrochloric acids in anhydrous methanol for 24 h at 80°C to obtain the methylglcosides. Then the monosaccharide composition was determined by gas chromatography (GC) after derivatization *via* the hexamethyl disilazane (HMDS) and the trimethylchlorsilane (TMS) reaction. The mannitol was added to the samples as the internal standard. Additionally, the presence of Fru was tested with the Urea-HCl colorimetric method (15).

The glycosidic linkages were determined by methylation. The carrier gas was Helium (pressure control: 80 kPa). The relative amount of each type of linkage was determined based on the area of each compound and related to the monosaccharide compositions of each fraction (16).

### 2.7. The NMR spectroscopy

After three deuterium exchanges using freeze-drying in D_2_O (10 mg/mL) and performance on a Bruker AV600 instrument (Bruker, Rheinstetten, Germany) at 25°C, the ^1^H NMR and ^13^C NMR spectra of SMP-NP and SMP-AP were recorded on the spectrometer (600 MHz). These peaks were labeled using MestReNova software (Version 6.0.2-5475, 2009, Mestrelab Research S.L., Spain).

### 2.8. Prebiotic effect

#### 2.8.1. *Lactobacillus* bacterial strains

The *Lactobacillus buchneri* (BSS1, CCTCC No. AB2016284), *L. johnsonii* (BS15, CCTCC: M2013663), *L. plantarum* (BS10, CCTCC: M012487), *L. plantarum* (BSGP201683, CCTCC: M2016425) and *L. rhamnosus* GG (LGG, ATCC53103) were obtained from professor Xue-Qin Ni of Animal Microecology Institute, College of Veterinary Medicine, Sichuan Agricultural University, China. *L. johnsonii* (Hjg8, ATCC 33200) was provided by Dr. Bing-zhao Zhang of Shenzhen Institutes of Advanced Technology, Chinese Academy of Science, China. All these were stored at −80°C in MRS medium with 20% glycerin.

#### 2.8.2. Bacterial growth

The basal medium (10 g peptone and tryptone, respectively, 5 g yeast extract, 1 mL of Tween 80, 0.5 g L-cysteine hydrochloride, 1 g/L carbohydrate source, and 1 L of distilled water with an adjusted pH at 6.5) and the MRS medium were used as culture mediums after being autoclaved at 121°C for 30 min. Two commercial prebiotic products were used as a positive control compared with SMP-NP. These products were P95s (96.1% fructo-oligosaccharides, DPn 2–9, with 2.7% glucose, fructose, and sucrose, the product of the partial enzymatic hydrolysis of chicory inulin) and Orafti^®^HP (99.8% inulin, DPav ≥23, with 0.2% glucose, fructose, and sucrose). Both were obtained from Quantum Hi-Tech (China) Biotechnology Co., Ltd., Shenzhen, China. These five strains of *Lactobacilli* were incubated in the 50 mL MRS medium at 37°C overnight in anaerobic chamber (Thermo Scientifific 1,029, in 5% N_2_, 10% H_2_, 5% CO_2_), then centrifuged 3,500 rpm, 10 min, and resuspended in saline and basal medium, successively, to remove the carbon source. Finally, they were resuspended with basal medium containing these three different carbon sources above (the CPPF and two commercially available prebiotic P95s, Orafti^®^HP), at a concentration of 10^7^–10^8^ CFU/mL, after adjusted by McIntosh Turbidimetric tube. 5 milliliter bacterial suspensions were divided in test tubes, and then incubated for 0 and 24 h. All test tubes were set in triplicate. Two hundred microliter of the basal medium was added to the 96-wells plates and the density of bacteria were measured at the wavelength of 600 nm (A600) using Multiscan Spectrum (Thermo Scientifific, Varioskan Flash) after incubated for 0 h and 24 h. The bacterial growth was evidenced as the increment in A600 (ΔA600) during 24 h of incubation in anaerobic chamber. After 24 h of incubation, the pH was measured by pH meter (A115200, Lichen Instrument technology Co. Ltd., Hunan, China) after removing bacteria by centrifuging at 4000 rpm for 20 min. Each tube was tested three times, triplicate each time, making sure high accuracy and precision (11).

### 2.9. Anti-oxidant activity determination

#### 2.9.1. Cell culture

IPEC-J2 cell (Intestinal porcine epithelial cell lines) was obtained from and maintained in DMEM/F-12 medium (Beijing Solarbio Science & Technology Co., Ltd.). This was supplemented with 10% FBS (Thermo Fisher Scientific (China) Co., Ltd), 100 U/mL penicillin, and 100 U/mL streptomycin (Beijing Solarbio Science & Technology Co., Ltd.) in a humidified atmosphere of 5% CO_2_ at 37°C.

#### 2.9.2. Establishment of oxidative stress damages model

IPEC-J2 cells were seeded into 96-well plates at a density of 1.0 × 10^4^ cells/well. After the cells were adhered in the 96-well plate, the culture medium was washed with a phosphate buffer saline (PBS, pH = 7.4, Beijing Solarbio Science & Technology Co., Ltd.) three times. 200 μmol/mL of H_2_O_2_ (Sigma-Aldrich, United States) were added into the 96-well plate (*n* = 12) and cultured in a 37°C incubator. After 24 h incubation, 10 μL of CCK8 (Wuhan Boster Biological Technology., LTD, Wuhan, China) was added to the 96-plate wells. After a 1 h incubation, the measurement was performed at 450 nm with a microwell reader (Bio-Rad).

#### 2.9.3. Measurement of IPEC-J2 cells viability

IPEC-J2 cells were seeded into 96-well plates and cultured in a 37°C incubator for 24 h. 200 μmol/mL of H2O2 was added to the plate and cultured for 24 h. Three concentrations of SMP-AP (20 μg/mL, 10 μg/mL, 5 μg/mL) were added to the 96-well plate. After being cultured at 37°C for 24 h, the cell viability was determined by the CCK8 method.

#### 2.9.4. Determination of antioxidant enzymes activity

IPEC-J2 cells were seeded into a 6-well plate, and an oxidative stress damages model was established using 200 μmol/mL of H_2_O_2_. Different concentrations of SMP-AP (20 μg/mL, 10 μg/mL, 5 μg/mL) were added to the six-well plate. After being cultured at 37°C for 24 h, plates were washed with PBS three times. Cells were collected by a cell culture dish and disrupted by a cell ultrasonic cell breaker (Shanghai Huxi Industry Co., Ltd). The cells were then centrifuged at 12,000 rpm for 3 min after cell disruption to obtain the supernatant for the determination of the antioxidant enzyme activity. The antioxidant enzyme activities were determined by a Biochemical Detection Kit (Nanjing Jiancheng Bioengineering Institute, Nanjing, China).

#### 2.9.5. Quantitative real-time PCR

RNA extraction from the intestine cells and real-time PCR for antioxidant gene detection were performed as previously reported (17). In summary, IPEC-J2 cells were lysed with Trizol Regent (R1100, Beijing Solarbio Science & Technology Co., Ltd., China), and the total RNA was extracted from the cells according to the manufacturer’s instructions. The RNA quality was determined by the agarose gel method, and the RNA concentration was determined by a spectrophotometer (NanoDrop 2000, Shanghai Institute of Thermal Sciences, China). The total RNA was reverse transcribed using reverse transcriptase which was done according to the manufacturer’s instructions (Enzyme Tower, Waltham, Mass.). The Bio-Rad-CFX96 system was used for real-time quantitative PCR, and related gene expression was normalized with the internal control β-actin. The primer sequences of the SYBR green probe of the target gene are shown in Table 2.

**Table 2:** qRT-PCR primers for antioxidant defense genes.

Gene	bp	Primer sequence
Nrf2	125	F: CACCACCTCAGGGTAATA
		R: GCGGCTTGAATGTTTGTC
NQO1	200	F: GATCATACTGGCCCACTCCG
		R: GAGCAGTCTCGGCAGGATAC
HO-1	130	F: AGCTGTTTCTGAGCCTCCAA
		R: CAAGACGGAAACACGAGACA
CAT	124	F: TGTGAACTGTCCCTTCCGTG
		R: CGTCTGTTCGGGAGCACTAA
SOD1	176	F: ACCTGGGCAATGTGACTG
		R: TCCAGCATTTCCCGTCT
GPXs	127	F: CGGACCACCTGTTGAAAGCTC
		R: TCCGCCAGTTCTTGTTGTCCA
ZO-1	126	F: TTGATAGTGGCGTTGACA
		R: CCTCATCTTCATCATCTTCTAC
β-Actin	122	F: GATGAGATTGGCATGGCTTT
		R: CACCTTCACCGTTCCAGTTT

### 2.10. Statistical analysis

The statistical values were represented as mean ± SD. The statistical comparisons were applied with the one-way analysis of variance (ANOVA) by Duncan’s test using SPSS version 20.0. Then the values of *p* < 0.05 and *p* < 0.01 were considered statistically significant and highly significant, respectively.

## 3. Results

### 3.1. Optimization of extraction process of Shenmai polysaccharide

#### 3.1.1. Effects of temperature, solvent/material ratio, and extraction time on the yield of SMP

**Figure 1:**
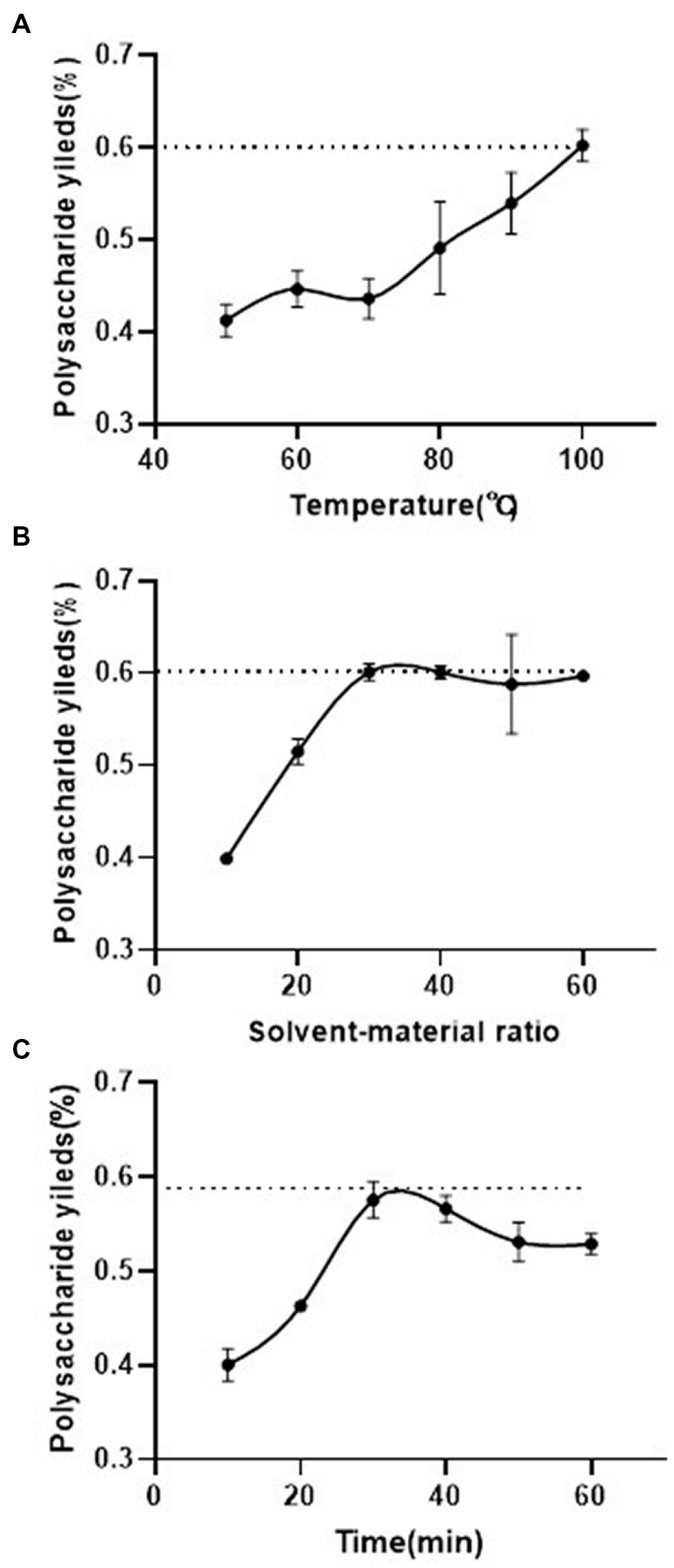
Effect of different extraction parameters on the yield of polysaccharide from Shenmai injection waste. **(A)** Extraction temperature (the S/M ratio and extraction time were fixed at 30 mL/g and 30 min, respectively); **(B)** solvent/material ratio (the extraction temperature and time were fixed at 100°C and 30 min, respectively); **(C)** extraction time (the extraction temperature and S/M ratio were fixed at 100°C and 30 mL/g, respectively). Values are the means of polysaccharide yield (*n* = 3).

Optimized single-factor extraction experiments were performed to better isolate polysaccharides from the Shenmai injection waste (named Shenmai polysaccharide, SMP). As shown in Figure 1A, the extraction yield of SMP increased as the extraction temperature increased (*p* < 0.05), showing a linear relationship. When the water extraction temperature reached 100°C, the extraction yield of polysaccharide reached its maximum amount (60.25%) (Figure 1A). Therefore, 90°C was chosen as the center level of extraction temperature in the response surface design. The other two temperature points were set at 80°C and 100°C. With the temperature at 100°C and an extraction time of 30 min, the extraction yield reached 58.80% when the solvent/material ratio was around 30 mL/g (Figure 1B). But when the solvent/material ratio was increased from 30 mL/g to 50 mL/g, the extraction yield was 60.75%, only increasing 1.95% (Figure 1B, *p* > 0.05). Thus, 30 mL/g was set as the central value in the response surface design. The other two values were set as 20 mL/g and 40 mL/g, respectively. With the temperature at 100°C and a solvent/material ratio of 30 mL/g, it was found that the polysaccharide extraction yield increased with increased extraction time, reaching a maximum value (56.79%) around 30 min (Figure 1C). Therefore, 30 min was set as the center point for extraction time. The other points for extraction time were set at 20 min and 40 min.

#### 3.1.2. Optimization of extraction yield using RSM

##### 3.1.2.1. Model fitting

SAS. JMP13.0 software was used to carry out a regression analysis of the data in Table 1. This provided the following predicted regression equation of the three factors corresponding to the yield of SMP:


$$\begin{array}{c} Y=4.4978X_{1}+3.1491X_{2}+3.0244X_{3}-0.0194X_{1}X_{2}\\ -0.0145X_{1}X_{3}-0.0202X_{2}X_{3}-0.0178X_{1}^{2}\\ -0.0091X_{2}^{2}-0.0155X_{3}^{2}-252.6963 \end{array}$$

*X*_1_, extraction temperature; *X*_2_, solvent/material ratio; *X*_3_, extraction time.

##### 3.1.2.2. Analysis of response and contour surface plots

Variance analysis was conducted on the model (Table 3). It was found that the *R*^2^ of this model was 0.97 (*p* = 0.0023 < 0.01), and the multiple regression relationship between dependent variables and all independent variables was significant (Table 3). The primary term *X*_1_ (extraction temperature) of the model was extremely significant (*p* < 0.001), suggesting that extraction temperature had the greatest impact on the yield of SMP.

**Table 3:** Regression coefficients for three dependent variables.

Regression coefficients	*p*
*X*_1_	0.00077^c^
*X*_2_	0.00119^b^
*X*_3_	0.00461^b^
*X*_1_*X_1_	0.03764^a^
*X*_1_*X_2_	0.01440^a^
*X*_1_*X_3_	0.04273^a^
*X*_2_*X_2_	0.16370
*X*_2_*X_3_	0.01269^a^
*X*_3_*X_3_	0.03764^a^

*X*_1_, extraction temperature; *X*_2_, S/M ratio; *X*_3_, extraction time.

a Significant at 0.05 level.

b Significant at 0.01 level.

c Significant at 0.001 level.

##### 3.1.2.3. Optimization of extraction conditions

**Figure 2:**
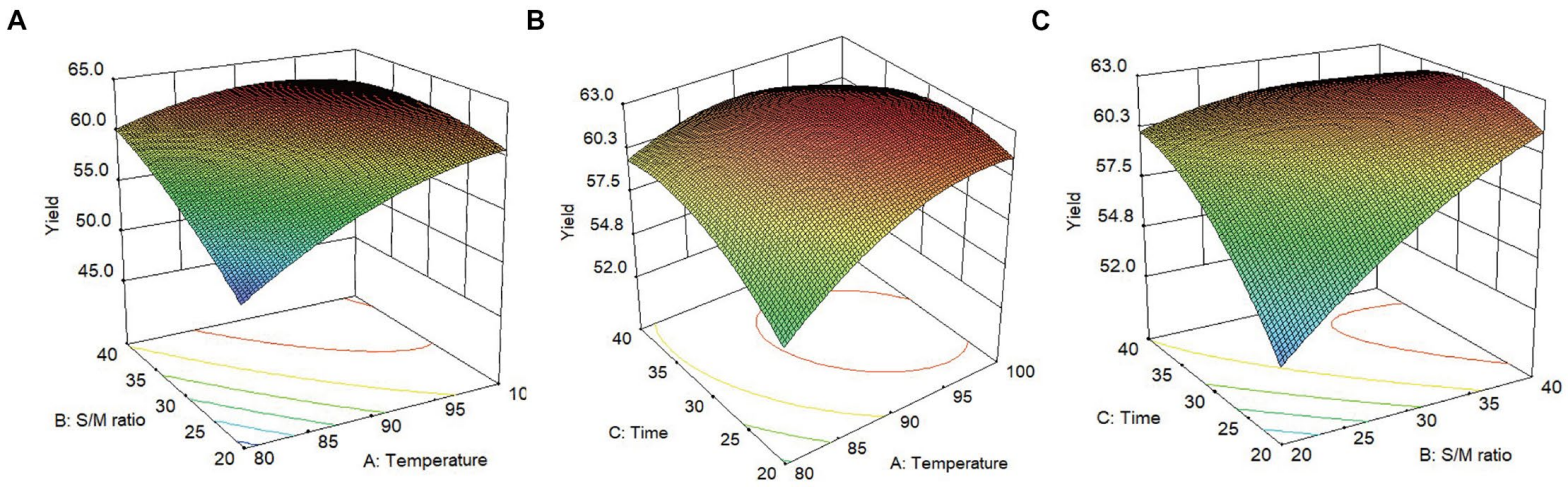
Effect of three factors interacting on the extraction yield of Shenmai polysaccharide (left, response surface plots; right, contour plots); **(A)** effect of extraction temperature (*X*_1_) and solvent-material ratio (*X*_2_) on the yield of Shenmai polysaccharide; **(B)** effect of extraction temperature (*X*_1_) and extraction time (*X*_3_) on the yield of Shenmai polysaccharides; **(C)** effect of solvent-material ratio (*X*_2_) and extraction time (*X*_3_) on the yield of Shenmai polysaccharides.

Obtained through the software’s statistical analysis, the response surface figure is shown in Figure 2. It can be seen that the yield of SMP was greatly affected by the above three factors. This was consistent with the regression coefficient results in Table 1. When the extraction time was fixed at the 0 level, the yield of SMP increased with the increase of extraction temperature (*X*_1_, 80°C–97.70°C) and the solvent/material ratio (20–40 mL/g) (Figure 2A). However, when the temperature is higher than 97.70°C, the yield of SMP decreases again. When the solvent/material ratio is fixed at the 0 level, the yield of SMP increased as the extraction temperature (*X*_1_, 80°C–97.70°C) and extraction time (20–35.92 min) increased (Figure 2B). When the extraction temperature was fixed at the 0 level, the yield of SMP increased with the increase of the ratio of solvent/material (20–40 mL/g) and extraction time (20–35.92 min). When the extraction time exceeds 35.92 min, the yield of SMP decreased (Figure 2C). In addition, it can be seen from Figure 2 that the following pairs all have a linear relationship with the yield of SMP: the extraction temperature (*X*_1_) and the solvent/material ratio (*X*_2_), the extraction temperature (*X*_1_) and the extraction time (*X*_3_), and the solvent/material ratio (*X*_2_) and the extraction time (*X*_3_). This is similar to the analysis results in Table 3. In summary, extraction temperature, solvent/material ratio, and extraction time all have a certain impact on the yield of SMP. The order of the three factors was extraction temperature, extraction time, and then solvent/material ratio.

##### 3.1.2.4. Verification of the models

The fitting equation obtained in 3.1.2.1 was analyzed by JMP software. Based on the results, we concluded that when the extraction temperature was 93.10°C the solvent/material ratio was 40 mL/g, the extraction time was 27.94 min, and the yield of SMP reached its maximum value of 62.84%. To ensure convenient production, the optimal extraction process conditions were determined. They are as follows: an extraction temperature of 93°C, a solvent-material ratio of 40 mL/g, an extraction time of 30 min each time, extraction twice, and a predicted extraction rate of 62.78%.

To verify the accuracy of the above fitting model, the SMP was extracted twice for 30 min using the optimal extraction process. The final yield of SMP was 62.72%, which was close to the predicted value (62.78%), indicating a good fit with the above regression equation. This extraction process can accurately display the trend of SMP production. It meets the requirements of both experimental and actual production.

### 3.2. Chemical composition of crude Shenmai polysaccharide

The protein, polyphenols, and carbohydrate content in crude polysaccharides were determined. Crude SMP was 81.89% carbohydrates, 8.82% protein, and 2.31% polyphenols. Therefore, purification processes were performed to obtain purified Shenmai polysaccharides.

### 3.3. Purification of polysaccharide

**Figure 3:**
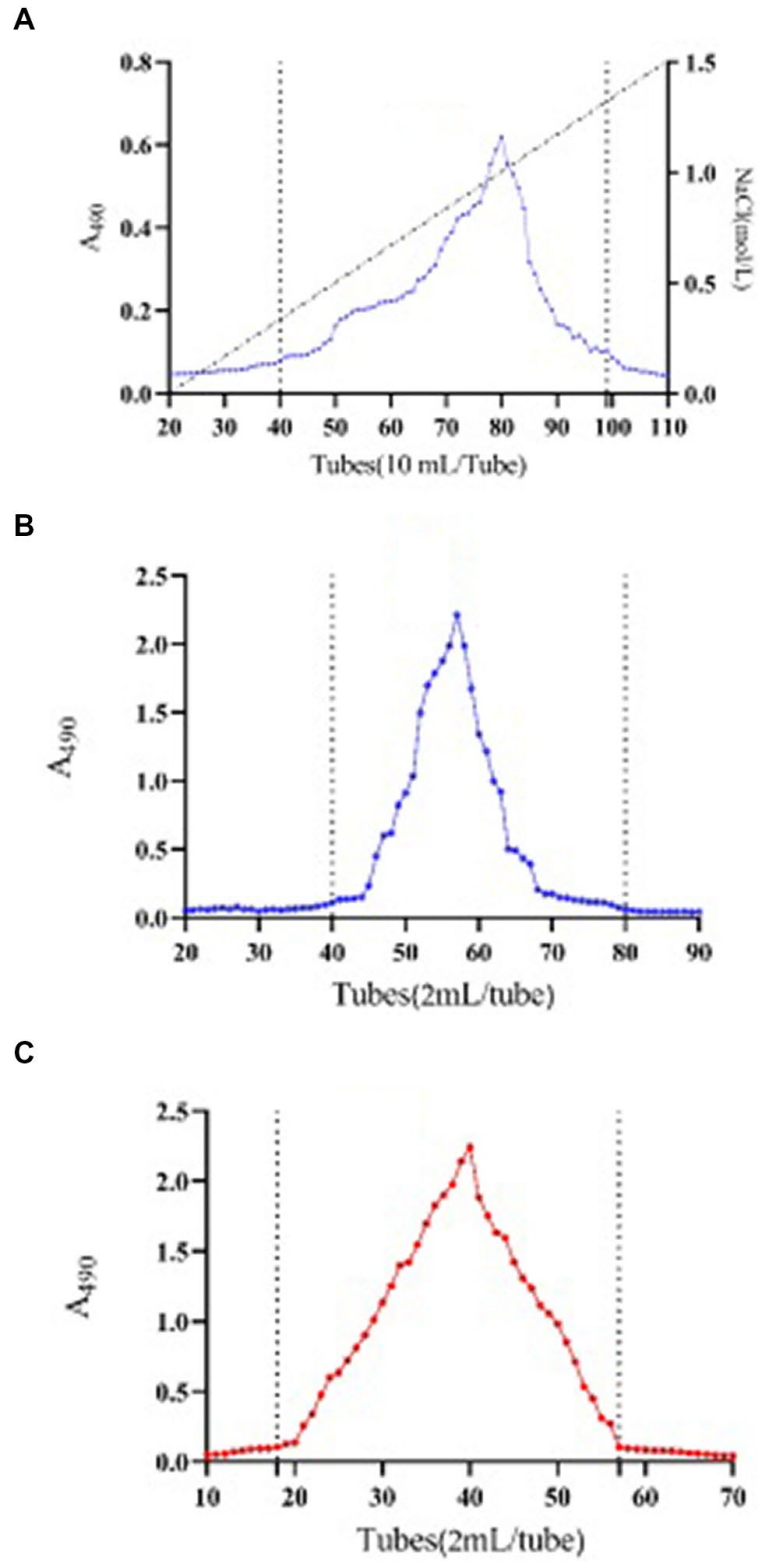
Purification elution profiles. DEAE anion exchange chromatography elution file of Shenmai polysaccharide **(A)**. Size exclusion chromatography elution profile of fraction SMP-NP **(B)** and SMP-AP **(C)**. A490 stands for absorbance at 490 nm as described in phenol-sulphuric acid method.

The neutral polysaccharide of SMP was eluted by distilled water. 238.8 mg of neutral polysaccharide fraction was obtained from 310.7 mg of crude SMP, named SMP-NP. An acidic polysaccharide fraction with a mass of 15.63 mg was collected by DEAE anion exchange chromatography, named SMP-AP (Figure 3A). This data indicated that neutral polysaccharides (yield 76.86%) account for the majority of the SMP. To obtain the purified polysaccharide fraction, gel filtration was performed. One fraction was obtained with symmetrical and uniform curves for both SMP-NP (Figure 3B) and SMP-AP (Figure 3C), indicating that these two polysaccharides are homogeneous components.

### 3.4. Molecular weight and monosaccharide composition determination

The molecular weight of the SMP-NP and SMP-AP were determined by size exclusion chromatography. The results showed that the molecular weight of SMP-NP and SMP-AP were 30.8 kDa and 256.7 kDa, respectively.

The results of the monosaccharide composition determination of these two polysaccharide components are shown in Table 4. There are two monosaccharide components in SMP-NP, glucose, and fructose. It has a glucose content of 10.1%, and the molar ratio of fructose to glucose is about 9:1. SMP-AP consists of five monosaccharide components: glucose, galactose, galacturonic acid, rhamnose, and arabinose. It has a molar ratio of 95:2.0:1.6:0.8:0.5.

**Table 4:** Monosaccharide composition determination results of SMP-NP and SMP-AP.

Sample	Monosaccharide composition	Mol %
SMP-NP	Glc	10.1
	Fru	89.9
SMP-AP	Glc	95.0
	Gal	2.0
	GalA	1.6
	Rha	0.8
	Ara	0.5

### 3.5. Determination of glycosidic linkage

#### 3.5.1. Glycosidic linkage units of SMP-NP

The results of the glycosidic linkage determination of SMP-NP are shown in Table 5. SMP-NP is mainly composed of Fru and Glc, which is consistent with the results of monosaccharide composition determination. The linkage units in Fru are mainly 1,2-Fru*f* and 1,2,6-Fru*f*, and these have a molar ratio of 53.0% and 27.4%, respectively. In addition, terminal Fru*f* is also presented in SMP-NP and has a molar ratio of 8.5%. The linkage units in Glc are 1,4-Glc*p*, 1,6-Glc*p,* and terminal Glc*p*. These contain a molar ratio of 8.8%, 0.6%, and 1.6%, respectively.

**Table 5:** Glycosidic linkage units of SMP-NP.

Monosaccharides	Glycosidic bond	Mol %
Fru	T-Fru*f*	8.5
	1,2-Fru*f*	53.0
	1,2,6-Fru*f*	27.4
Glc	T-Glc*p*	1.6
	1,4-Glc*p*	8.8
	1,6-Glc*p*	0.7

#### 3.5.2. Glycosidic linkage units of SMP-AP

The glycosidic linkage unit determination results of SMP-AP are shown in Table 6. SMP-AP is composed of 1,4-GalA*p* and 1,4-Gal*p* linked units, and these have a molar ratio accounting for 51.1% and 8.6%, respectively. Rha in SMP-AP contains the linkage units 1,2-Rha*p* and 1,2, 4-Rha*p*, which have a molar ratio of 1.9% and 7.4%, respectively. At the same time, Ara in SMP-AP also showed two kinds of glycosidic units. T-Ara*f* was the main linkage unit and had a molar ratio of 6.1%. In addition to this, the Glc in SMP-AP is mainly connected by 1,3-Glc*p*.

**Table 6:** Glycosidic linkage units of SMP-AP.

Monosaccharides	Glycosidic units	Mol %
Rha	1,2-Rha*p*	1.9
	1,2,4-Rha*p*	7.4
Glc	T-Ara*f*	6.1
	1,5-Ara*f*	0.9
GalA	T-GalA*p*	1.5
	1,4-GalA*p*	51.1
Glc	1,3-Glc*p*	0.94
Gal	1,4-Gal*p*	8.6

### 3.6. NMR analysis

#### 3.6.1. NMR analysis of SMP-NP

**Figure 4:**
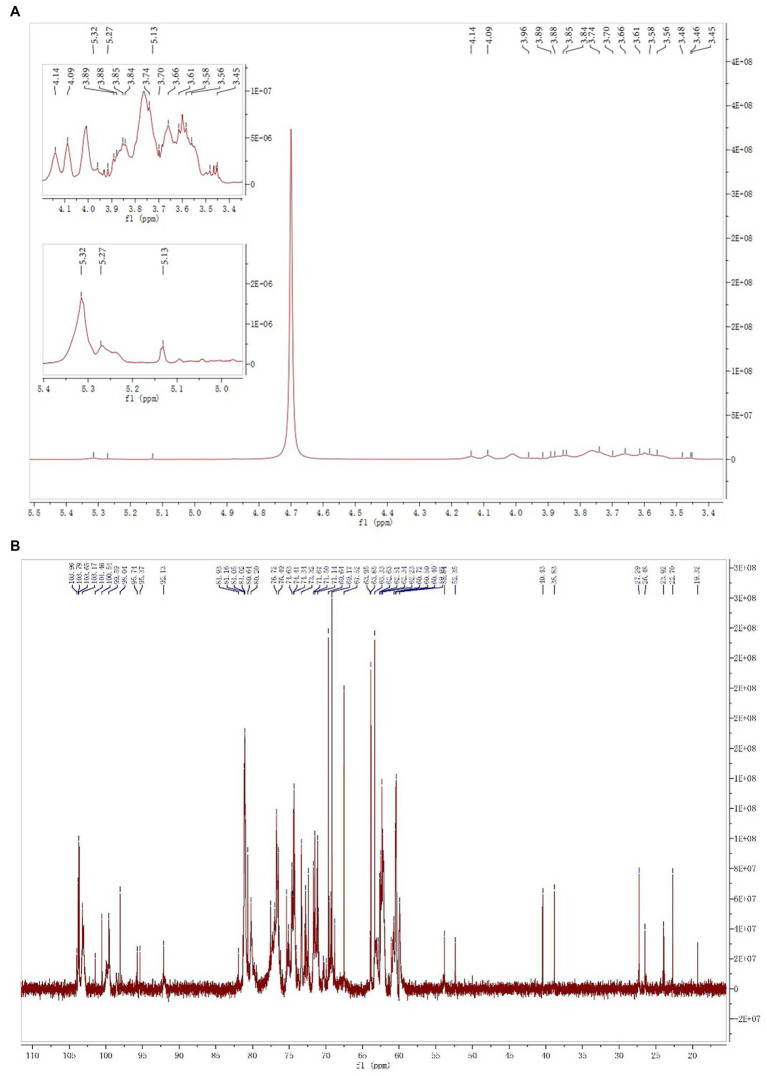
The 1D NMR spectrum of SMP-NP in D_2_O **(A)** ^1^H NMR spectrum; **(B)** ^13^C NMR spectrum.

The ^1^H spectrum of SMP-NP is shown in Figure 4A, and the ^13^C NMR spectrum is shown in Figure 4B. ^13^C-NMR results show that the carbon signal concentrated in δ 102–104 ppm assigned itself to the C-2 signal peak of the Fru*f* residue. Three of the most obvious signal peaks were δ 103.79, δ 103.65, and δ 103.17 ppm. According to previous reports, the heterocephalic carbon signals are δ 62.63 ppm, δ 60.50 ppm, and δ 60.40 ppm, respectively. The corresponding proton signals are δ 3.85, δ 3.84, and δ 3.66 ppm, respectively. This indicates that the three Fru*f* residues in SMP-NP are β-configuration (18–20). The heterocephalic carbon signals concentrated in δ 92–101 ppm should be attributed to Glc residues. The heterocephalic carbon signals of the three signal peaks were δ 92.13, 98.04, and 99.58 ppm, respectively. Combined with the results of ^1^H spectrum, which has the H-1 signals at δ 5.27, 5.31, and 5.13 ppm, respectively, the three residues proved to be α-D-Glc*p* configuration (21–29). The specific chemical shifts of ^1^H and ^13^C of the above sugar residues are shown in Table 7. Based on the results of all the above SMP-NP structures, SMP-NP seemed to be a mixed fructan composed of Fru and Glc. 1,2-β-D-Fru*f*, 1,2,6-β-D-Fru*f,* and T-β-D-Fru*f* were its main backbone and are connected with the α-D-(1 → 4)-Glc*p*, α-D-(1 → 6)-Glc*p* and T-Glc*p* residues.

**Table 7:** ^13^C and ^1^H chemical shifts (ppm) of polysaccharide fraction SMP-NP.

Residues	C-1/H-1	C-2/H-2	C-3/H-3	C-4/H-4	C-5/H-5	C-6/H-6
1 → 2-β-D-Fru*f*	62.63/3.85	103.17	76.49/4.27	74.63/4.14	81.05/3.88	63.85/3.85
1,2,6-β-D-Fru*f* T-β-D-Fru*f*	60.50/3.84	103.65	76.72/4.14	75.02/4.09	80.97/3.61	62.34/3.74
	60.40/3.66	103.79	76.71/4.09	74.67/4.01	81.16/3.76	63.33/3.58
1 → 4-α-D-Glc*p*	99.58/5.31	74.63/3.56	74.41/4.01	80.20/3.70	71.14/4.29	60.4/3.85
1 → 6-α-D-Glc*p*	98.04/5.13	71.50/3.61	74.41/3.76	69.64/3.58	69.17/3.96	63.95/3.92
T-α-D-Glc*p*	92.13/5.27	71.14/3.45	73.21/3.76	74.34/3.89	74.63/3.74	60.72/3.66

#### 3.6.2. NMR analysis of SMP-AP

**Figure 5:**
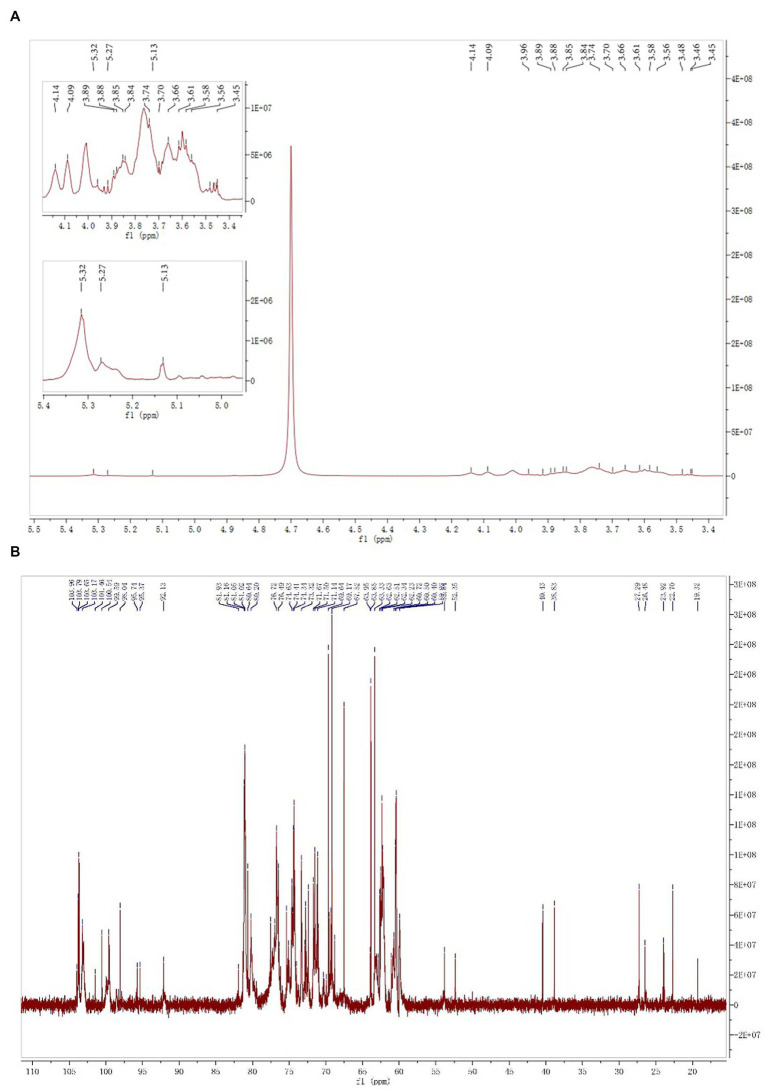
The 1D NMR spectrum of SMP-NP in D_2_O **(A)** ^1^H NMR spectrum; **(B)** ^13^C NMR spectrum.

The ^1^H spectrum of SMP-AP is shown in Figure 5A, and the ^13^C spectrum is shown in Figure 5B. The signals of δ 17.69 ppm and δ 94.89 ppm in ^13^C-NMR spectra were assigned to C-6 and C-2 of Rha*p*. The signals of δ 1.31 ppm and δ 5.30 ppm were assigned to H-6 and H-2 of Rha*p*. Taken together, those results indicated the presence of α-1,2-Rha*p* in SMP-AP (30, 31). The signals δ 170–173.55 ppm were assigned to C-6 of carboxyl signal peaks. This indicated that SMP-AP contains Gal*p*A residues. The chemical shift of H-1 was δ 4.57 ppm, and the chemical shift of C-1 was δ 98.76 ppm. This suggested that the residue is α-D-GalA*p*. In addition, ^13^C-NMR results showed that the H-1 proton signals of Ara were mainly concentrated around δ 5.0–5.25 ppm, while the C-1 carbon signals were mainly concentrated around δ 107 − 112 ppm (31–37). According to the literature, the signal at δ 5.14 ppm was assigned to H-1 of α-D-Gal*p*, and the C-1 carbon signal of this residue was at δ 102.01 ppm (38).

Based on the above structural analysis of SMP-AP, the main backbone of SMP-AP was α-(1 → 3)-Glc*p*, which is a typical glucan structure. At the same time, α-(1 → 4)-GalA and α-L-(1 → 4)-Rha*p* are on the main chain of SMP-AP, which is a typical RG-I pectin structure. In addition, there were Ara*f* and Gal*p* residues on the 2^nd^ position of Rha*p* as branch chain connections. So, we speculated that there were a few arabinogalactan residues in SMP-AP. We concluded that SMP-AP is an acidic polysaccharide composed of glucan and RG-I pectin that has a small amount of arabingalactan linked to it as branched chains (39).

### 3.7. Prebiotic activity

Five *Lactobacilli* strains were cultured in a medium with different carbon sources, and their density was evaluated after 48 h. As shown in Table 8, the bacterial density and the bacterial density increment of the SMP-NP group were significantly higher than those in the saline group (*p* < 0.05). This indicated that five different *Lactobacilli* strains can ferment and utilize SMP-NP as a carbon source to support their proliferation *in vitro*. We then compared this with Orafti^®^HP (DPn ≥ 23). When p95s (DPn 2–9) was used as a carbon source, four *Lactobacilli* strains showed better growth and greater changes in bacterial density. This suggested that P95s were an easily used carbon source for these four different *Lactobacilli* strains. The effect of SMP-NP on the proliferation of the five different *Lactobacilli* strains was similar to that of P95s.

**Table 8:** Capacity to ferment SMP-NP or commercial prebiotics by *Lactobacilli* strains.

*Lactobacillus strain*	P95s	Orafti^®^HP	SMP-NP	Glc	Saline	MRS
*L. plantarum*X3	0.38 ± 0.03^cB^	0.22 ± 0.06^dC^	0.42 ± 0.07^dB^	0.48 ± 0.02^eB^	0.12 ± 0.00^bD^	1.94 ± 0.03^aA^
*L. johnsonii* BS15	0.59 ± 0.01^bC^	0.63 ± 0.05^bC^	0.6 ± 0.06^cC^	0.83 ± 0.00^bB^	0.02 ± 0.00^dD^	1.44 ± 0.02^dA^
*L.plantarum*BS10	0.59 ± 0.03^bC^	0.35 ± 0.02^cD^	0.66 ± 0.01^bC^	1.1 ± 0.00^aB^	0.04 ± 0.00^dE^	1.86 ± 0.03^bA^
*L. buchneri* BSS1	0.18 ± 0.00^dC^	0.13 ± 0.00^eC^	0.17 ± 0.00^eC^	0.73 ± 0.01^cB^	0.07 ± 0.01^cD^	1.93 ± 0.03^aA^
*L. rhamnosus*lgg	0.73 ± 0.12^aB^	0.69 ± 0.03^aB^	0.77 ± 0.04^aB^	0.6 ± 0.05^dC^	0.22 ± 0.07^aD^	1.97 ± 0.00^aA^

A vertical comparison of five different *Lactobacilli* strains showed that SMP-NP could promote the proliferation of five different *Lactobacilli* strains *in vitro*, but the extent to which the five *Lactobacilli* strains used SMP-NP was different. The greatest fermentation utilization rate of SMP-NP was by *L. johnsonii* BS15, and its bacterial density changed the most after 48 h of fermentation, reaching 30 times that of the saline group (*p* < 0.05). The utilization rate of SMP-NP by *L. plantarum* BS10 was also very good, and its bacterial density reached 16.5 times that of the saline group after 48 h of fermentation. *L. buchneri* BSS1 utilization rate was relatively poor, and the bacterial density after 48 h was about twice that of the saline group.

As shown in Table 9, five different strains of *Lactobacilli* were cultured in an anaerobic environment and they each displayed different pH values in the culture medium. The pH values of the five media supplemented with SMP-NP decreased in varying degrees. In addition, the pH values of the media supplemented with MRS were significantly decreased. However, in the medium supplemented with SMP-NP, there was no significant difference in the degree of pH reduction among the five *Lactobacilli* strains. The decrease in pH was due to the metabolites produced by *Lactobacilli*, such as lactic acid, acetic acid, and other types of short fatty acids. The increase in bacterial density and lower pH indicated the growth of probiotics and the effective use of SMP-NP.

**Table 9:** The final pH of medium containing SMP-NP or commercially prebiotics by *Lactobacilli* strains.

*Lactobacillus strain*	P95s	Orafti^®^HP	SMP-NP	Glc	Saline	MRS
*L. plantarum*X3	5.4 ± 0.00^bB^	5.93 ± 0.06^aA^	5.3 ± 0.00^bB^	5 ± 0.00^bB^	6.2 ± 0.00^bA^	4.5 ± 0.10b^cC^
*L. johnsonii* BS15	5.4 ± 0.00^bB^	5.33 ± 0.06^dB^	5.17 ± 0.06^bB^	5 ± 0.10^bB^	6.17 ± 0.06^bA^	4.33 ± 0.15^cC^
*L.plantarum*BS10	5.37 ± 0.06^cB^	5.63 ± 0.06^cB^	5.33 ± 0.12^bB^	4.87 ± 0.06^c^	6.27 ± 0.06^abA^	4.53 ± 0.06^bC^
*L. buchneri* BSS1	5.62 ± 0.06^aB^	5.67 ± 0.06^cB^	5.67 ± 0.15^aB^	5.43 ± 0.06^aB^	6.33 ± 0.06^aA^	5 ± 0.10^aC^
*L. rhamnosus*lgg	5.2 ± 0.17^dC^	5.77 ± 0.12^bB^	5.7 ± 0.00^aB^	5.33 ± 0.06^aC^	6.3 ± 0.00^aA^	4.87 ± 0.06^aD^

### 3.8. Anti-oxidant activity

**Figure 6:**
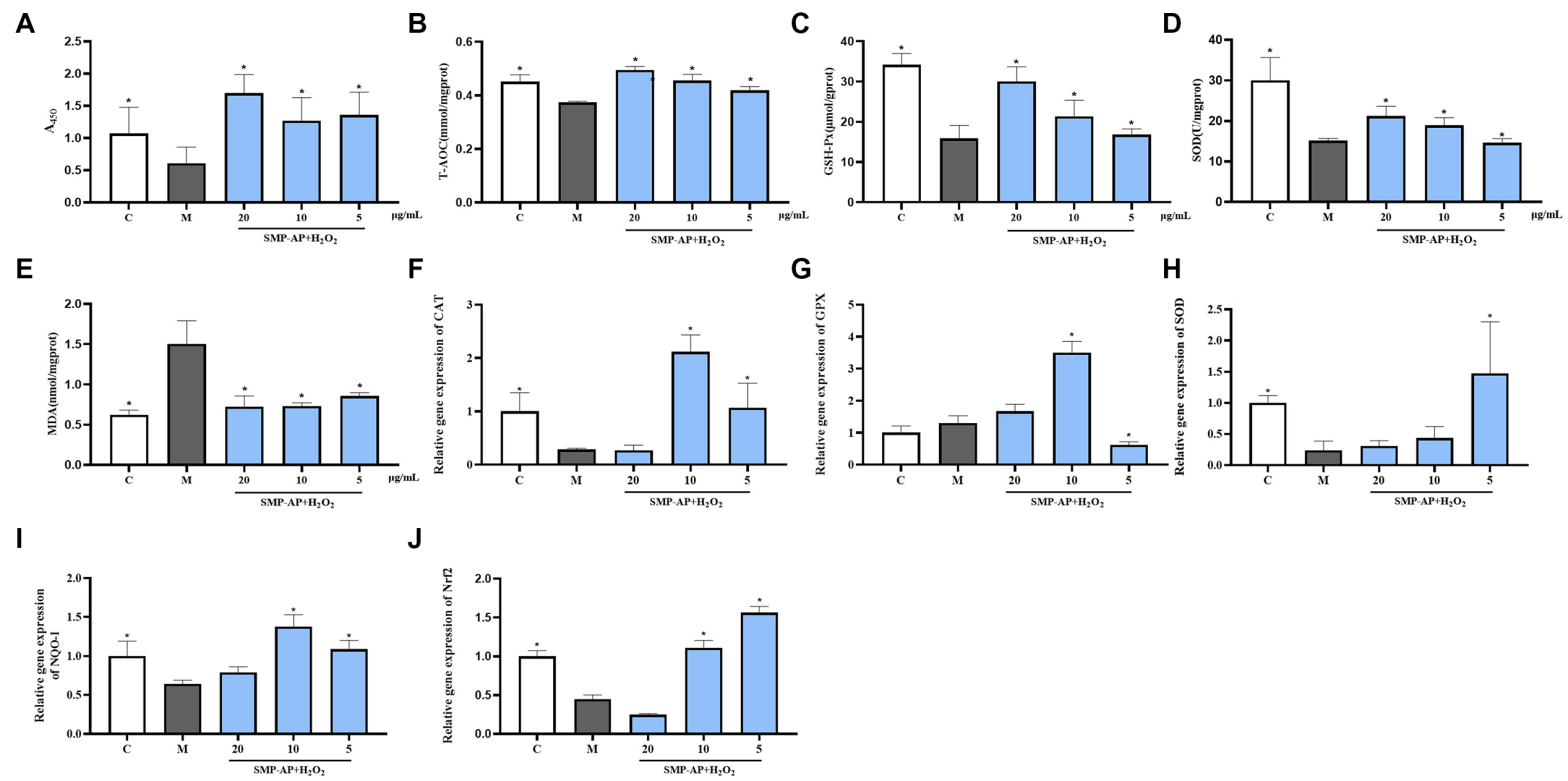
SMP-AP promote antioxidant defense of IPEC-J2 cells. **(A)** Quantification illustrates the cell viability of cells treated with 200 μmol/mL H_2_O_2_ for 24 h and then with different concentrations of SMP-AP for 24 h. **(B)** Quantification illustrates the level of T-AOC. **(C–E)** Quantification illustrates the activity of GSH-Px, SOD and the level of MDA. **(F–J)** qRT-PCR illustrates mRNA levels of CAT, GPX, SOD, NQO1 and Nrf2 in IPEC-J2 cells.

To evaluate the antioxidant effect of SMP-AP on intestinal epithelial cells, a porcine jejunal epithelial cell line (IPEC-J2) treated with H_2_O_2_ was used as an *in vitro* model. The results showed that H_2_O_2_ treatment significantly inhibited the cell viability of IPEC-J2 cells and that SMP-AP could protect IPEC-J2 cells from this defect (Figure 6A). Further biochemistry measurements showed that in the group supplement with SMP-AP, total antioxidant capacity (T-AOC), glutathione peroxidase activity (GSH-Px) and superoxide dismutase activity (SOD) increased. However, lipid oxidation products—MDA decreased, indicating that the protective effect of SMP-AP on oxidative stress may be due to it mediating the cellular antioxidant defense of IPEC-J2 cells (Figures 6B–E). To analyze the regulatory mechanisms of SMA-AP in cellular antioxidant defense, we quantified the expressions of genes associated with these processes. First, we noted that in H_2_O_2_-treated IPEC-J2 cells supplied with SMA-AP, expressions of some critical antioxidant genes were significantly increased (Figures 6C,F–I). These include antioxidant genes like catalase (CAT), glutathione peroxidase (GPX) and superoxidase dismutase (SOD), NQO-1. We concluded that this expression increase was responsible for increased cellular antioxidant defense activity. Secondly, we quantified the expression of the key transcriptional factor –Nrf2, a direct regulator of these antioxidant genes. In doing so, we found that SMA-AP could also enhance the expression of Nrf2 in H_2_O_2_-treated IPEC-J2 cells (Figure 6J). In summary, our results indicated that SMA-AP could be used as an effective component to treat oxidative stress-related defects by regulating cellular antioxidant defense.

## 4. Discussion

### 4.1. Optimization of extraction process of polysaccharide from Shenmai injection waste by response surface methodology

*Panax ginseng* and *O. japonicus* are both traditional Chinese medicinal materials. Polysaccharide is one of the main active components of these two herbs and has been researched before. However, only a few studies focused on extracting SMP from the waste of the Shenmai injection. Through the optimal extraction conditions, 62.72% polysaccharide was obtained in this study. This proved that there were a large number of polysaccharide components in the production waste of the Shenmai injection, which needed further development and utilization.

Polysaccharide is a natural product and is supplied by several sources. Naturally, the technology used to extract it has always been the focus of research. Recently, there have been many reports on the extraction technology of polysaccharides from *P. ginseng* or *O. japonicus.* A study was conducted to investigate the effects of temperature, solid-material ratio, and extraction time on the yield of *P. ginseng* (40). The results showed that the optimal extraction process was as follows: the liquid–solid ratio was 12:1, the extraction time was 3.5 h, the extraction temperature was 100°C, and it was extracted twice, the yield of polysaccharides from *P. ginseng* reached 22% under these conditions. However, another study showed that the optimal extraction process was when the ratio of material to liquid was 1:8, water bath extraction was used 3 times at 100°C and for 3 h each time (41). And yet, another study had optimum extraction conditions that put the extraction temperature at 100°C, extraction time at 4 h, and the liquid–solid ratio at 15:1 (42). In our study, the optimal extraction temperature of SMP was 93°C, the extraction time was 27.94 min, and the ratio of solvent to the material was 40 mL/g. These large differences in optimization may be due to different material treatment methods. Most of the Chinese medicinal materials in the other studies were extracted by direct shearing, while in our study, the two medicinal materials were ground into powder for extraction. Many studies have shown that the effective components in plant cells can be dissolved only after infiltration, swelling, more infiltration, and then diffusion. However, the pulverization of medicinal materials can significantly improve the wall-breaking rate of plant cells, thereby improving the dissolution of effective components (43, 44). This is likely the reason why we were able to have a high extraction rate of SMP in a short amount of time in this study. On a different note, other studies showed that the optimal water extraction process of *O. japonicus* polysaccharide was to extract twice at 100°C for 2 h each time and to keep a liquid to material ratio of 6:1. The order of factors affecting the water extraction of *O. japonicus* polysaccharide was said to be: extraction time > extraction temperature > ratio of liquid to material > extraction times (45). However, the results of this study showed that the most influential factors on the polysaccharide were extraction temperature, extraction time, and then the ratio of solvent to material. The above differences may have been caused by the variety of medicinal materials, harvest time, and polysaccharide content (46). Additionally, there are significant differences in the extraction rate and polysaccharide content of different herb medicines, so the combined extraction of red *P. ginseng* and *O. japonicus* may also be the reason for the differences in the extraction process between studies.

### 4.2. Isolation, purification, and structure elucidation of Shenmai polysaccharide

The structure of polysaccharides is closely related to biological activity. Polysaccharides with different structures have different pharmacological activities. The molecular weight is related to the advanced structures formed by the polysaccharides. Polysaccharide GRS1-I with a molecular weight of 4.611 kDa was obtained from *P. ginseng* by the method of amylase and alcohol precipitation (47). But another polysaccharide, this one with a molecular weight of 1.5 kDa, was obtained from *P. ginseng* by ethanol precipitation at different concentrations (41). The molecular weights of those two polysaccharides were much smaller than SMP-NP and SMP-AP. The different extraction conditions may be the reason for the differences in their molecular weights. However, another neutral polysaccharide fraction named, PGPW1, was isolated from *P. ginseng* with a *Mw* of 350 kDa. This was higher than other reported *P. ginseng* polysaccharides (48). Regarding the polysaccharides from *O. japonicus*, there were several studies reported, such as MD-1, MD-2, OJP1, and OJP1 ~ 4 that had *Mw* ranging from 2.70 kDa to 324.65 kDa (28, 47). These polysaccharides were significantly different from the *Mw* of the two polysaccharide fractions obtained in the present study. This may be because the polysaccharides obtained in our study were isolated from a mixture of *P.gingseng* and *O. japonicus* rather than from a single plant.

Studies have shown that the polysaccharides in Chinese patent medicine may come from a single medicine and that new polysaccharides may be produced during the extraction process. Our results showed that the molar ratio of Fru and Glc was 35:1 and that the main connection between Fru was 2 → 1 or 2 → 6, which was very similar to the SMP-NP structure. Several studies were able to obtain a similar structure of polysaccharides from *O. japonicus*, but the molar ratio of Fru to Glc were 30:1 (49), 15:1 (50) and 12:1 (51). WGPA-N and WGPN are neutral polysaccharides in *P. ginseng* that were eluted by distilled water (52). The study found that those two neutral polysaccharides were composed of Glc, Gal, and Ara. The study also found that the molar ratio of these three monosaccharides was 3.3:95.3:1.3 and 18.0:66.3:15.7, respectively. Glc was mainly connected as 1 → 4 or 1 → 6, which was similar to how Glc connected in SMP-NP. Two other researchers extracted two neutral polysaccharides from *P. ginseng*, using different methods (40), and found that those neutral polysaccharides not only contained Glc, Gal, and Ara but also held a small amount of mannose. The main linkage units of Glc were 1 → 4 linked Glc, with a small amount of 1 → 6 linked Glc, and the results were similar to SMP-NP. In summary, Fru in SMP-NP may come mainly from *O. japonicus*, while Glc may come mainly from *P. ginseng*. Some studies have shown that when the production process of polysaccharides is different, some polysaccharide components may change. These changes include things like losing a monosaccharide component or having inconsistent molar ratios of the monosaccharide (53). This may be the reason why SMP-NP develops different monosaccharide molar ratios with *P. ginseng* and/or *O. japonicus* between studies.

Acidic polysaccharides are polysaccharides with carboxyl groups. Most of the acidic polysaccharides that came from *P. ginseng* are pectic polysaccharides. Fewer studies reported acidic polysaccharides coming from *O. japonicus*. SMP-AP is an acidic polysaccharide composed of GalA, Gal, Ara, Glc, and Rha. This is very similar to the structure of the *P. ginseng* acidic polysaccharide that has been previously published (54–56). It shows that in the acidic polysaccharide S-A-I from *P. ginseng*, with a Gal residue connected by 1 → 6 GaL and further connected by 1 → 5 or 1 → 3, five Ara and GalA residues were presented as 1 → 4 linked GalA. This is very similar to how the monosaccharides in SMP-AP are connected. The difference is that these *P. ginseng* acidic polysaccharides do not contain Glc. The results of the structural elucidation of WRGP indicated that the main component of WRGP was RG-I pectin and that it contained more of the AG type side chain. However, unlike SMP-AP, WRGP also contained manose. When isolated from *P. ginseng*, GPW-1, and GPW-2 have monosaccharide compositions similar to those of SMP-AP (54). Another study showed that all six acidic pectins from *P. ginseng* have glycosidic linkages. This is also similar to SMP-AP (57). Therefore, due to the similarity of glycosidic linkages and monosaccharide compositions, it is possible that the acidic polysaccharide of Shenmai injection waste mainly comes from *P. ginseng*. However, SMP-AP and the above-mentioned pectin showed different monosaccharide compositions and molar ratios. This may be due to the differences in materials, extraction, and purification methods, all of which can lead to changes in the monosaccharide composition.

### 4.3. Prebiotic activity

Prebiotics refer to organic substances that are not digested and absorbed by the host. Instead, they selectively promote the metabolism and proliferation of beneficial bacteria in the body, thereby improving the host’s health (58). Polysaccharides are one of the most common prebiotics. In this study, the density of multiple strains of *Lactobacilli* was measured after 48 h of incubation. The degree of proliferation over 48 h reflected the degree of carbon source utilization. The results showed that five different *Lactobacilli* strains could ferment and utilize SMP-NP as a carbon source. This increased bacterial density, indicating that SMP-NP could promote the proliferation of these five different *Lactobacilli* strains *in vitro*. At the same time, as the density of bacteria increased, their metabolites increased, such as lactic acid and acetic acid (59, 60). This resulted in a decrease in the pH of the culture medium. After measuring the pH values of different *Lactobacilli* strains cultured on different media, it was found that the medium supplement with SMP-NP could get a pH value much lower than the saline group. In summary, SMP-NP can promote the proliferation of five *Lactobacillus* strains and reduce the pH of the culture medium, meaning it has potential prebiotic activity.

### 4.4. Anti-oxidant activity

Oxidative stress and disruption of the intracellular redox balance have been identified as the key potential factors in the progression of animal diseases (61). With this in mind, SOD and CAT are important antioxidant enzymes. SOD plays a crucial role in balancing oxidative and antioxidant effects. It is a free radical scavenger that can scavenge superoxide anion radicals. Additionally, the high and low viability of SOD indirectly reflects the body’s ability to scavenge free radicals (62). CAT is a ubiquitous enzyme that efficiently promotes the decomposition of H_2_O_2_ into H_2_O and O_2_ to prevent cellular oxidative damage (63, 64). MDA is the final product of these oxygen-free radicals’ lipid peroxidation (65). The level of MDA content indirectly reflects the severity of the free radicals attacks on body cells.

In the past several decades, numerous natural polysaccharides and fructans have been shown to have significant antioxidant activity using different evaluation methods (60). Our results indicated that SMP-AP exhibited significant antioxidant activity. The SMP used in this study were extracted from a combination of red *P. ginseng* and *O. japonicus*. However, numerous studies have shown that *P. ginseng* polysaccharides can significantly increase the levels of the antioxidant enzymes SOD, CAT, and GPX-Px, as well as the non-enzymatic compound reduced glutathione (GSH). These studies also showed that *P. ginseng* polysaccharides can decrease malondialdehyde (MDA) levels against oxidative stress (66). The Shengmai injection was also investigated and had similar antioxidant activity, which was consistent with our study (67). However, the mechanism of the antioxidant activity of polysaccharides is still unknown, which need further study in future.

## 5. Conclusion

In this study, the optimal extraction conditions for crude polysaccharides from Shemmai injection waste were obtained by RSM. When the extraction temperature was at 93°C, the ratio of solvent to material was 40 mL/g, and the extraction time was 30 min, the maximum yield of SMP was 62.72%. After purification, a neutral fraction (SMP-NP) and an acidic fraction (SMP-AP) were obtained. The neutral fraction was a levan, and the acidic fraction was a pectic polysaccharide. SMP-NP could be fermented by five strains of Lactobacillus. It was able to reduce the pH of the culture medium, and it may be a potential prebiotic. The acidic polysaccharide SMP-AP exhibited potential antioxidant activity *in vitro*. All these results suggested that, due to its polysaccharides, Shemmai injection waste could be used to develop potential prebiotics and anti-oxidants.

## Data availability statement

The original contributions presented in the study are included in the article/supplementary material, further inquiries can be directed to the corresponding author.

## Author contributions

JK and Z-KZ: conceptualizaltion, methodology, validation, and investigation. JZ: software. Z-KZ: formal analysis and writing—original draft preparation. L-XL: resources. JK and JZ: data curation. JZ, L-FH, and M-LT: writing—review and editing. JK: visualization. L-XL and M-LT: supervision, project administration, and funding acquisition. All authors contributed to the article and approved the submitted version.

## Funding

This study was funded by National Natural Science Foundation of China, grant number 82004041.

## Conflict of interest

The authors declare that the research was conducted in the absence of any commercial or financial relationships that could be construed as a potential conflict of interest.

## Publisher’s note

All claims expressed in this article are solely those of the authors and do not necessarily represent those of their affiliated organizations, or those of the publisher, the editors and the reviewers. Any product that may be evaluated in this article, or claim that may be made by its manufacturer, is not guaranteed or endorsed by the publisher.
